# Effectiveness of Outpatient Chronic Pain Management for Middle-Aged Patients by Internet Hospitals: Retrospective Cohort Study

**DOI:** 10.2196/54975

**Published:** 2024-12-30

**Authors:** Ling Sang, Bixin Zheng, Xianzheng Zeng, Huizhen Liu, Qing Jiang, Maotong Liu, Chenyu Zhu, Maoying Wang, Zengwei Yi, Keyu Song, Li Song

**Affiliations:** 1 Department of Pain Management, West China Hospital, Sichuan University, No 37 Guoxue Xiang, Wuhou District, Chengdu, 610041, China, 86 18980601501; 2 Center of Biostatistics, Design, Measurement and Evaluation (CBDME), Department of Clinical Research Management, West China Hospital, Sichuan University, Chengdu, China

**Keywords:** chronic pain management, internet hospital, physical hospital, quality of life, outpatient care, telemedicine, digital health

## Abstract

**Background:**

Chronic pain is widespread and carries a heavy disease burden, and there is a lack of effective outpatient pain management. As an emerging internet medical platform in China, internet hospitals have been successfully applied for the management of chronic diseases. There are also a certain number of patients with chronic pain that use internet hospitals for pain management. However, no studies have investigated the effectiveness of pain management via internet hospitals.

**Objective:**

The aim of this retrospective cohort study was to explore the effectiveness of chronic pain management by internet hospitals and their advantages and disadvantages compared to traditional physical hospital visits.

**Methods:**

This was a retrospective cohort study. Demographic information such as the patient’s sex, age, and number of visits was obtained from the IT center. During the first and last patient visits, information on outcome variables such as the Brief Pain Inventory (BPI), medical satisfaction, medical costs, and adverse drug events was obtained through a telephone follow-up. All patients with chronic pain who had 3 or more visits (internet or offline) between September 2021, and February 2023, were included. The patients were divided into an internet hospital group and a physical hospital group, according to whether they had web-based or in-person consultations, respectively. To control for confounding variables, propensity score matching was used to match the two groups. Matching variables included age, sex, diagnosis, and number of clinic visits.

**Results:**

A total of 122 people in the internet hospital group and 739 people in the physical hospital group met the inclusion criteria. After propensity score matching, 77 patients in each of the two groups were included in the analysis. There was not a significant difference in the quality of life (QOL; QOL assessment was part of the BPI scale) between the internet hospital group and the physical hospital group (*P*=.80), but the QOL of both groups of patients improved after pain management (internet hospital group: *P*<.001; physical hospital group: *P*=.001). There were no significant differences in the pain relief rate (*P*=.25) or the incidence of adverse events (*P*=.60) between the two groups. The total cost (*P*<.001) and treatment-related cost (*P*<.001) of the physical hospital group were higher than those of the internet hospital group. In addition, the degree of satisfaction in the internet hospital group was greater than that in the physical hospital group (*P*=.01).

**Conclusions:**

Internet hospitals are an effective way of managing chronic pain. They can improve patients’ QOL and satisfaction, reduce treatment costs, and can be used as part of a multimodal strategy for chronic pain self-management.

## Introduction

Chronic pain is one of the leading causes of disability worldwide, affecting approximately 30% of the population [1] and more than 300 million people in China [2]. Chronic pain can be considered a disease rather than a symptom [3], and its prevalence is higher among women, older individuals, workers, and people with low income and education levels [1,4]. Patients with chronic pain often experience psychological problems, such as anxiety and depression. Additionally, they often experience physical problems, such as loss of work ability and social withdrawal [1]. At the same time, chronic pain also places an enormous burden on the social economy, not only because of the direct cost of medical treatment but also because of the indirect cost of labor loss. According to a 2010 study, the United States spends $560‐635 billion annually on chronic pain [1]. In 2020, China spent 500 billion yuan (US $72.08 billion) on chronic pain [2].

The current treatment for chronic pain is based on multimodal and biopsychosocial models [5]. Despite a variety of treatments, chronic pain is undertreated. In China, only 14.3% of the population considers chronic pain to be a disease [6], and most people misunderstand pain treatment and lack knowledge on pain self-management [7]. According to Notaro et al [8] and Gregory and McGowan [9], in patients who were hospitalized, pain was reported in 50%, chronic pain was reported in 21.7%, and acute exacerbation of chronic pain was reported in 70%. Thus, chronic pain affects 1 in 5 people inside the hospital, let alone outside the hospital.

According to a survey in 15 European countries, 64% of patients who were using medication reported that their pain medication was insufficient to control their pain, and the majority of respondents had never been treated by a pain specialist [10]. Zheng et al [11] reported that 36.8% of patients had never received pain-related treatment. Similarly, Li et al [12] reported that 40.8% of patients with chronic pain had not received drug treatment. At the same time, Schneider et al [13] reported that 18.6% of older patients receiving home care did not use pain medication, and patients who used pain medication reported deficiencies in its effectiveness. One of the factors that prevents patients from seeing a doctor outside the hospital is that pain specialist outpatient clinics are only available in economically developed areas and tertiary hospitals [7]. Based on the rapid change of pain conditions, determining how to dynamically manage pain is a problem for patients outside of the hospital [14].

Telemedicine and mobile health have been applied to pain management. In the beginning, telemedicine was mainly used for remote consultation, remote treatment, and first aid for the military, which later included families and individuals [15]. Mobile health mainly takes the form of health care apps [16]. Studies have shown that telemedicine and mobile health care can help patients manage pain [17-21]. With the development of internet technology and the further development of medical models, internet hospitals have emerged.

The concept of internet hospitals encompasses telemedicine, mobile health care, and internet health [22]. An internet hospital is a medical platform in which medical institutions use IT to extend hospital resources through the internet and provide medical services directly to patients [23]. In general, as a new derivative model, an internet hospital is more convenient than telemedicine, has a lower equipment demand, and has more powerful and reliable resources for doctors than mobile medicine. Therefore, internet hospitals may be more suitable for the management of chronic pain for outpatients. However, there has been little research on the ability of internet hospitals to manage chronic pain outside of hospitals.

At present, the term “internet hospital” mainly appears in China [24], as one of the forms of internet health care. There are minimal internet hospitals in internet health care outside of China, which mainly focuses on mobile medical apps, disease monitoring, electronic medical records, and other types of internet health care [25,26]. Internet hospitals have been successfully applied to manage chronic diseases, such as diabetes, hypertension, and COVID-19. During the COVID-19 pandemic, internet hospitals were conducive to alleviating social panic, preventing cross-contamination caused by crowd gatherings, and reducing incorrect medical-seeking behaviors [27]. Through the drug delivery platform of internet hospitals, relevant drugs can be delivered to patients without contact [28]. Research has shown that internet hospitals can improve patients’ self-management effectiveness for chronic diseases such as hypertension and diabetes [29-31]. We have described the development process and the classification and functions of internet hospitals in our previous study [24], and the internet hospitals involved in this study are all hospital-led and managed by designated tertiary hospitals.

Our previous research investigated the status of internet hospital use for patients with pain and revealed that 12.9% of patients used internet hospitals for pain management at home [24]. However, no further studies have explored the effectiveness of using an internet hospital for chronic pain management at home. Therefore, the aim of this retrospective cohort study was to compare the effects of using internet hospitals and physical hospitals (traditional in-person, outpatient follow-ups) for patients with chronic pain.

## Methods

### Study Design

This was a retrospective cohort study conducted from September 2021, to February 2023. Patients with ≥3 web-based consultations formed the internet hospital group; similarly, those with ≥3 in-person consultations formed the physical hospital group. Both internet hospitals and physical hospitals could provide patients with appointment registrations, prescriptions, drug adjustments, and other services. The West China Internet Hospital was the web-based platform of the West China Hospital of Sichuan University. The hospita linked patients with medical services through the two software platforms of the West China Hospital of Sichuan University, the WeChat official account and the Huayitong app (version 7.0.4), so that patients could have access to convenient, high-quality, and efficient professional medical services anytime and anywhere.

### Data Source

Patients with chronic pain who attended the outpatient clinic of the Pain Department of West China Hospital of Sichuan University from September 2021, to February 2023, were enrolled. The basic information of the patients, including age, sex, disease diagnosis, number of visits, visit time, consultation with an internet hospital or physical hospital, and contact information was obtained from the IT center of West China Hospital of Sichuan University. Then, we screened patients according to the inclusion and exclusion criteria, and propensity score matching (PSM) was performed for patients after screening according to sex, age, number of visits, and diagnosis. A telephone follow-up was conducted for the matched patients. The matched patients were asked to complete the Brief Pain Inventory (BPI) by telephone [32,33]. Moreover, the patients’ medical expenses, satisfaction, and occurrence of adverse drug events were also recorded by telephone follow-up.

### Participant Selection

The inclusion criteria were a pain duration or recurrence of ≥3 months, adults aged between 45 and 64 years, and ≥3 hospital visits. Patients with ≥3 pain clinic visits that were exclusively web-based formed the internet hospital group. Similarly, those with ≥3 pain clinic visits that were exclusively in-person formed the physical hospital group. The exclusion criteria were patients with mental illness; cognitive, vision, hearing, expression, or communication impairment; cancer pain; a history of drug abuse; addiction; alcohol abuse; and participation in any other clinical trial or study that affected pain intensity and quality of life (QOL).

### Outcome Variables

The primary outcome was improvement in QOL (QOL score at the last visit – the QOL score at the first visit). The secondary outcomes were patient satisfaction; pain relief rate, which was measured by the numerical rating scale (NRS; [NRS score at the first visit – the NRS score at the last visit]/NRS score at the first visit × 100); incidence of drug-related adverse events (stomach pain, severe constipation, dizziness, pruritus, nausea, and vomiting); and average cost per visit. The QOL score was measured by the QOL assessment part of the BPI. This part contained 7 items, including the impact of pain on patients’ daily life, mood, walking ability, daily work, relationship with others, sleep, and interest in life, with a score of 0‐10 points for each item. A lower score indicated a better QOL.

### Sample Size

At present, there are no studies related to outpatient pain management by internet hospitals, and there are no reference research data. Therefore, we conducted a preliminary experiment to calculate the required sample size. In the preliminary trial, 20 patients were included in both the internet hospital group and the physical hospital group according to the inclusion and exclusion criteria. The outcomes of these patients were obtained through telephone follow-up, and the sample size was calculated accordingly. The average difference between the QOL score at the last visit and the first visit was −4.35 (SD 6.12) in the internet hospital group and −9.65 (SD 8.41) in the physical hospital group. The α value was .05, and the β value was .9. The σ was the combined SD, and δ was the difference between the two groups of means. The calculation formula was n = (2[Z_α_ + Z_β_]^2^ × σ^2^)/δ^2^. Therefore, 56 patients were needed for each group, and considering a 10% loss rate, the final requirement was 64 patients per group.

### Statistical Analysis

All analyses were performed using SPSS 27.0 (IBM Corp). We matched the cohorts at a ratio of 1:1 using PSM. We used nearest neighbor matching with a caliper of 0.02, and the covariates were age, sex, disease diagnosis, and number of clinic consultations. For continuous variables such as age and number of visits, the standardized mean difference (SMD) was used to determine whether the match was balanced, with an SMD<0.10 indicating a balanced match [34]. For categorical variables, such as sex and diagnosis, we used an α level of .05 for the *χ^2^* test to indicate that the match was balanced. Means, SDs, medians, IQRs, and percentages were used to describe the data. A 2-tailed *t* test was used for normally distributed data, the Wilcoxon signed rank-sum test was used for nonnormally distributed data, and the *χ^2^* test or Fisher exact probability method was used for count data.

### Ethical Considerations

This study was approved by the Ethics Committee on Biomedical Research of the West China Hospital of Sichuan University (approval number: 2022 Review 467). The registration number in the Chinese Clinical Trial Registry was ChiCTR2200059152. Informed consent forms were signed by all patients who were followed up. The original data generated and analyzed in this study, such as the patient’s name, ID number, and home address, were anonymized. The study did not involve compensation for the enrolled patients.

## Results

### Participant Selection Process

**Figure 1.**
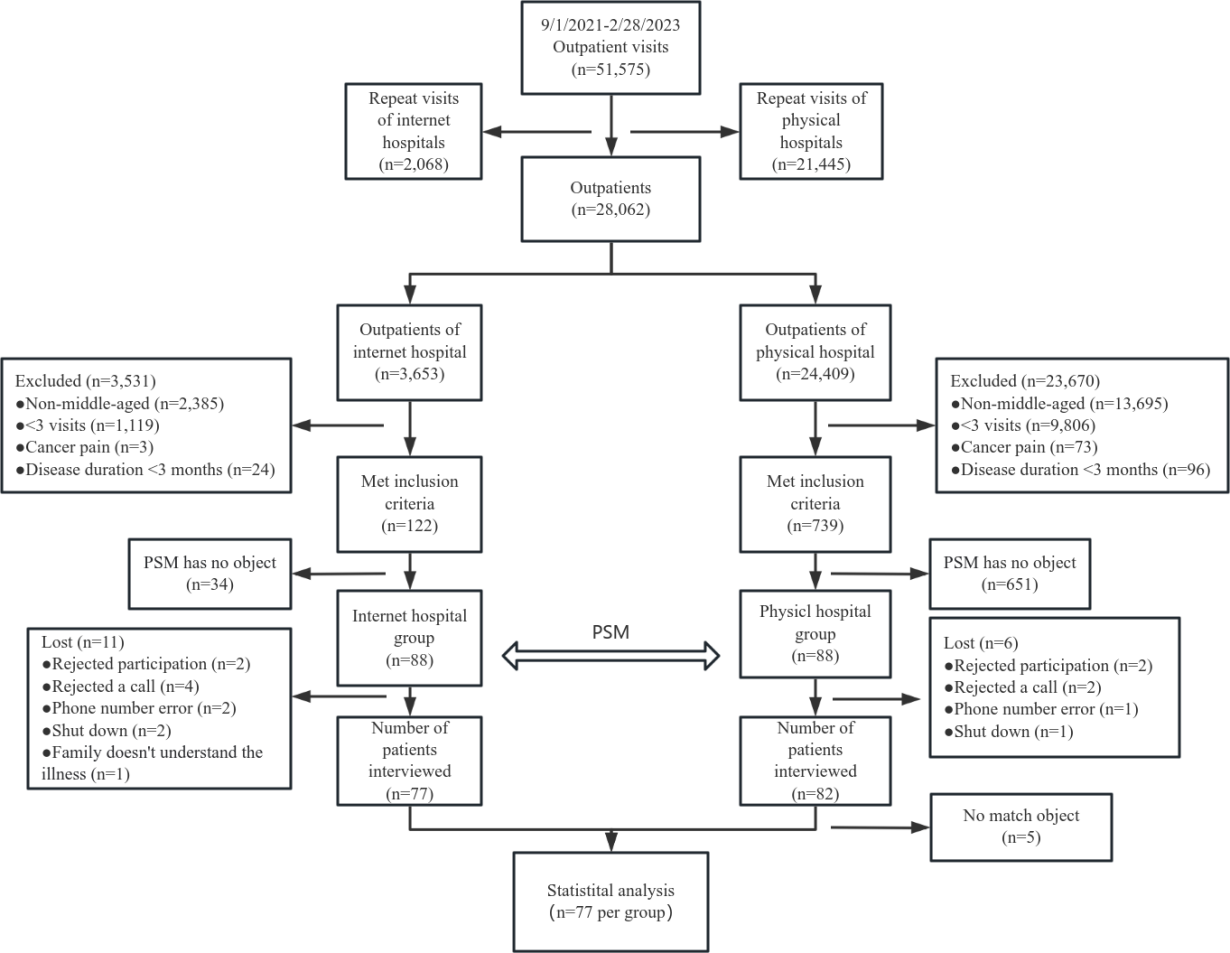
Flow chart of the sample selection process. PSM: propensity score matching.

The flow chart of the sample selection process is shown in Figure 1. From September 2021, to February 2023, there were a total of 51,575 outpatient visits. After excluding repeated visits, a total of 28,062 patients consulted the outpatient clinic during the day, including 3653 patients in the internet hospital group and 24,409 patients in the physical hospital group. After applying the inclusion and exclusion criteria, 3531 patients in the internet hospital group were excluded, and 23,670 patients in the physical hospital group were excluded. After 122 patients in the internet hospital group and 739 patients in the physical hospital group underwent PSM, 88 patients in each group were followed up by telephone. Of these patients, 11 were lost to follow-up in the internet hospital group, while 6 patients were lost to follow-up in the physical hospital group. Finally, both the internet hospital group and the physical hospital group included 77 patients for analysis.

### Characteristics of the Two Groups

Before the PSM was performed, there was not a significant difference in sex, age, or number of visits between the internet or physical hospital groups, but there was a significant difference in diagnosis (*P*<.001). Only significant differences in specific diseases that persisted after matching are shown. After PSM, there were no significant differences in sex, age, diagnosis, or number of visits between the two groups (Table 1). In addition, there were no statistically significant differences in education level, disease duration, or baseline QOL scores. The baseline NRS score of the internet hospital group was significantly higher than that of the physical hospital group (*P*<.001; Table 2). After PSM, a *P*>.05 and an SMD<0.10 for sex, age, number of visits, and disease diagnosis between the two groups indicated that the matching was balanced.

**Table 1.** Variables of the internet and physical hospital groups before and after propensity score matching.

Variables		Before matching (n=861)				After matching (n=154)			
		Internet hospital group(n=122）	Physical hospital group(n=739)	*P* value	SMD^a^	Internet hospital group(n=77)	Physical hospital group(n=77)	*P* value	SMD
Sex, n (%)				.24	0.12			>.99	0.00
Male		35 (28.7)	252 (34.1)			15 (19)	15 (19)		
Female		87 (71.3)	487 (65.9)			62 (81)	62 (81)		
Age (years), mean (SD)		54.44 (5.72)	54.72 (5.01)	.58	0.05	53.91 (5.72)	54.12 (4.83)	.81	0.04
Number of visits, median (IQR)		4.00 (4.00)	4.00 (2.00)	.71	0.04	4.00 (3.00)	4.00 (2.00)	.46	0.09
Disease diagnosis, n (%)				<.001	0.10			.99	0.04
Postherpetic neuralgia		12 (9.8)	97 (13.1)			9 (12)	10 (13)		
Osteoporosis		20 (16.4)	48 (6.5)			13 (17)	11 (14)		
Lumbar disc herniation		16 (13.1)	47 (6.4)			16 (21)	17 (22)		
Cervical disc herniation/cervical spondylosis		11 (9)	49 (6.6)			6 (8)	7 (9)		
Scapulohumeral periarthritis/rotator cuff injury		9 (7.4)	27 (3.6)			5 (6)	7 (9)		
Fibromyalgia		1 (0.8)	8 (1.1)			1 (1)	1 (1)		
Abdominal pain		3 (2.5)	9 (1.2)			3 (4)	2 (3)		
Headache		5 (4.1)	15 (2)			5 (6)	5 (6)		
Limb pain		9 (7.4)	26 (3.5)			8 (10)	5 (6)		
Pantalgia		10 (8.2)	20 (2.7)			10 (13)	10 (13)		
Perianal pain		2 (1.6)	4 (0.5)			1 (1)	2 (3)		
Other		24 (19.7)	389 (52.6)			—^b^	—^b^		

a SMD: standardized mean difference.

b Em dash: No other disease groups were found between the internet hospital group and physical hospital group after matching.

**Table 2.** Characteristics of the patients in the internet and physical hospital groups after propensity score matching.

Characteristics		Internet hospital group(n=77)	Physical hospital group(n=77)	Chi-square, *t* test, or W value (*df*)	*P* value
Education level, n (%)				5.695 (3)^a^	.13
Primary and below		36 (47)	27 (35)		
Junior high school		25 (32)	33 (43)		
Senior high school		2 (3)	7 (9)		
University and above		14 (18)	10 (13)		
Disease duration (month), median (IQR)		24 (36.00)	12 (48.00)	5770.5 (1, 152)^b^	.71
NRS^c^ score at baseline, mean (SD)		5.12 (1.522)	3.77 (1.376)	5.778 (152)^d^	<.001
QOL^e^ score at baseline, mean (SD)		12.84 (9.81)	15.27 (12.84)	−1.319 (152)^d^	.19

a Chi-square test.

b W value determined by a Wilcoxon signed rank-sum test.

c NRS: numerical rating scale.

d *t* test.

e QOL: quality of life.

### Quality of Life

**Figure 2.**
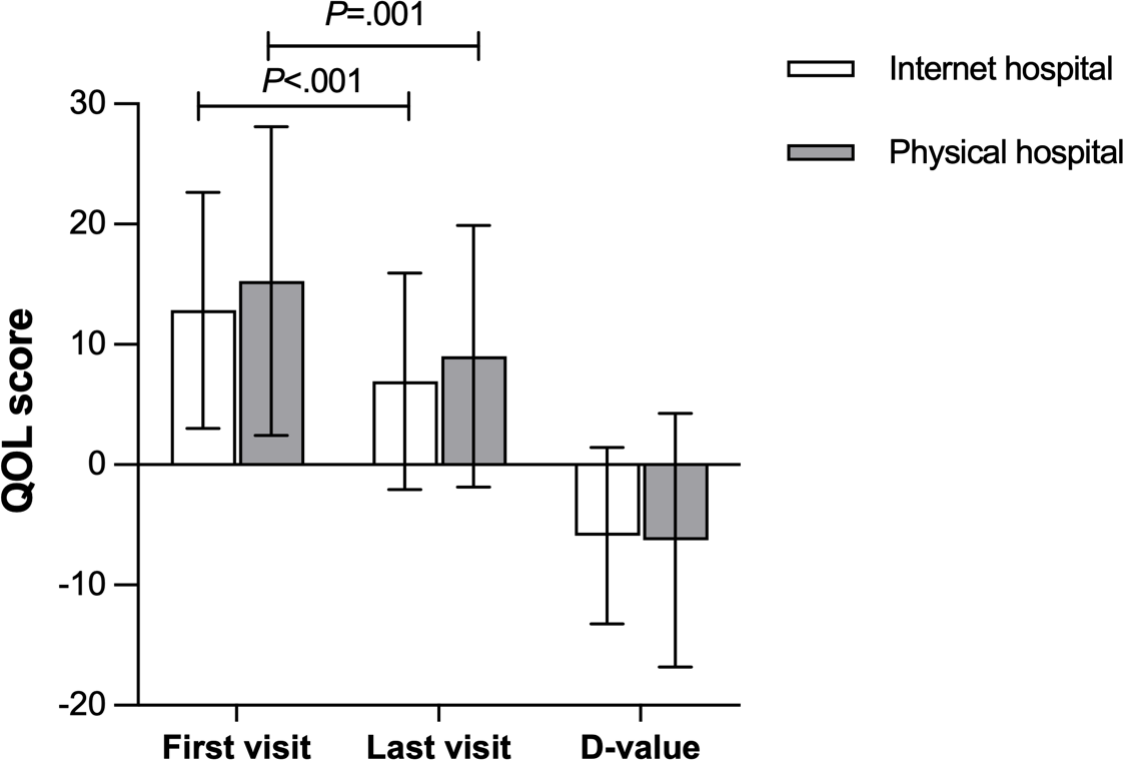
Quality of life scores for the internet and physical hospital groups at the first visit and last visit and their D-values. Error bars represent SD. D-value: quality of life at the last visit minus quality of life at the first visit; QOL: quality of life.

There was not a significant difference in the QOL score between the internet hospital group and the physical hospital group at the first visit (*P*=.19) or the last visit (*P*=.20). The difference in the QOL score between the last visit and the first visit was also not statistically significant between the two groups (*P*=.80; Figure 2). However, there was a significant decrease in the QOL score from the first visit to the last visit for the internet hospital group (*P*<.001). Similarly, there was a significant decrease in the QOL score from the first visit to the last visit for the physical hospital group (*P*=.001; Figure 2).

### Other Outcomes

**Figure 3.**
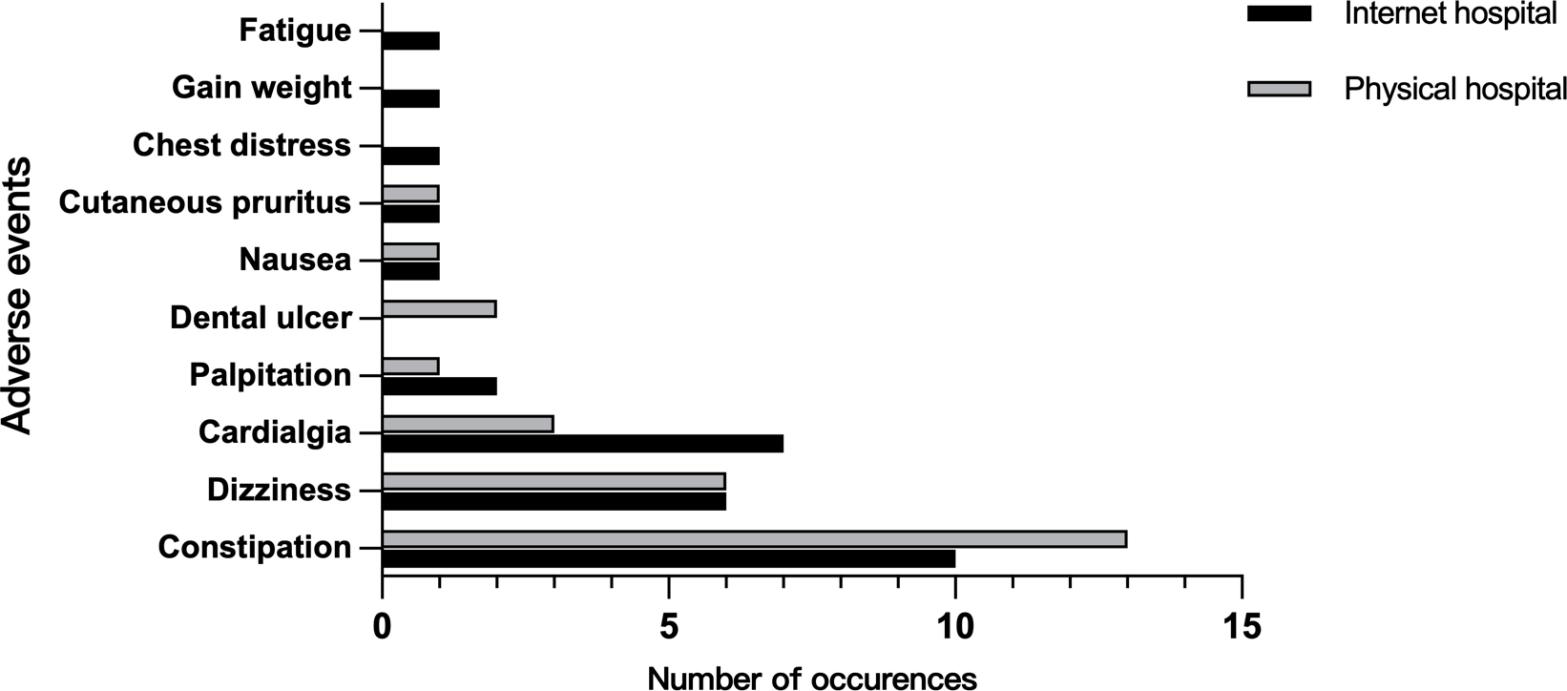
The number of adverse events.

There was not a significant difference in the pain relief rate (40.31% for internet hospital vs 34.11% for physical hospital *P*=.25) or the incidence of adverse drug events (26% for internet hospital vs 23% for physical hospital; *P*=.60) between the two groups, and the most common adverse events were constipation, dizziness, and cardialgia (Figure 3). In addition, there was a significant difference in patient satisfaction between the two groups (*P*=.01). More patients in the internet hospital group were very satisfied, and more patients in the physical hospital group were satisfied (Table 3).

**Table 3.** Comparison of other outcomes between patients of internet hospitals and physical hospitals.

		Internet hospital group(n=77)	Physical hospital group(n=77)	Chi-square, *t* test, or W value (*df*)	*P* value
Pain relief rate (%), mean (SD)		40.30 (29.87)	34.11 (35.60)	1.167 (152)^a^	.25
Adverse events, n (%)		26 (34)	23 (30)	0.269 (1)^b^	.60
Satisfaction, n (%)				10.989 (3)^b^	.01
Very satisfied		33 (43)	16 (21)		
Satisfied		29 (38)	44 (57)		
Uncertain		7 (9)	4 (5)		
Dissatisfied		8 (10)	13 (17)		
Very dissatisfied		0 (0)	0 (0)		
Total cost (yuan^c^), median (IQR)		130 (300.00)	410 (270.00)	4430.000 (1, 152)^d^	<.001
Medical expenses (yuan^c^), median (IQR)		130 (300.00)	300 (300.00)	4681.000 (1, 152)^d^	<.001

a *t* test.

b Chi-square test.

c A currency exchange rate of 1 yuan=US $0.13774 is applicable.

d W value determined by a Wilcoxon signed rank-sum test.

The median average cost per visit for patients in the internet hospital group was 130 yuan (US $17.87), while that in the physical hospital group was 410 yuan (US $56.35), and there was a significant difference between the two groups (*P*<.001). The expenses for the internet hospital group were all medicine-related expenses, while the physical hospital group also had travel, accommodation, and meal expenses in addition to medicine-related expenses. The median medical expense of patients in the physical hospital group was 300 yuan (US $41.23), which was significantly different from the 130 yuan (US $17.87) in the internet hospital group (*P*<.001; Table 3).

## Discussion

### Principal Findings

This was a retrospective cohort study that compared the effectiveness of chronic pain management between internet hospitals and traditional physical hospitals and revealed that both internet and physical hospitals could improve the QOL of patients with chronic pain. Patients using internet hospitals had a greater satisfaction and lower costs. However, there was no difference in the rate of pain relief and the incidence of adverse drug events between the two groups.

The distribution of medical resources is uneven in China, and high-quality medical resources are often concentrated in economically developed areas [35]. Research shows that the availability of medical resources has an impact on the use of mobile health care [36]. When visiting an outpatient clinic, patients have problems, such as long travel distances, difficult registration, long waiting times, and high costs, and one visit often cannot solve all of their health-related problems [37]. Patients with chronic pain need long-term multimodal pain management to relieve pain and reduce the occurrence of breakthrough pain.

Mobile medical apps for pain are currently in use, and some studies have shown that using mobile medical apps to manage pain can improve patients’ QOL and relieve pain [38-40]. For the management of chronic pain outside the hospital, mobile medical apps can be specially developed for a certain type of pain with high specificity, which can help patients with pain monitoring and evaluation, exercise rehabilitation, knowledge dissemination, and more. However, most mobile medical apps lack the participation of professional pain doctors and cannot provide patients with disease diagnosis, prescription issuance, drug adjustment, appointment booking, or other services. Moreover, mobile medical apps lack supervision to ensure their quality and accuracy and lack reliable evidence-based guidance on their use. In addition, the security of patients’ information is also a concern [16,41,42].

As a comprehensive platform, internet hospitals cover mobile medical apps and telemedicine. They rely on physical hospitals and can exercise some functions of physical hospitals. Internet hospitals can provide patients with services such as disease reviews, diagnosis and treatment, prescription issuance, and drug delivery. When patients’ pain worsens, they can make web-based appointments for offline diagnosis, treatment, examination, and hospitalization. More importantly, internet hospitals can also provide remote psychological care for patients, which is good for patient’s pain management [43]. To achieve the closed-loop management of patients inside and outside of the hospital, we described the functions of an internet hospital in our previous study [24].

Previous studies have shown that approximately 12.9% of patients choose internet hospitals for outpatient pain management, and the majority of patients in pain clinics are middle-aged patients (45‐64 years old) [24]. It is considered that older patients have problems with smartphone use and comprehension, and young patients mostly experience acute pain. This study was carried out among middle-aged patients. Considering the smooth communication with patients with cancer and their survival, they were excluded, as well as patients with mental illness or cognitive, vision, hearing, expression, or communication impairments.

In this study, patients’ QOL generally improved after outpatient pain management at both internet hospitals and physical hospitals. This finding is similar to those of several previous studies. Li et al [44] indicated that internet-based pain interventions for patients with chronic postoperative pain resulted in improvements in QOL and satisfaction similar to traditional face-to-face interventions. Buonanno et al [17] showed that telemedicine could provide high-quality assistance for patients with cancer-associated pain similar to face-to-face visits. Therefore, internet hospitals are effective ways to improve the QOL of patients with chronic pain, similarly to physical hospitals.

Patients in both groups received the guidance of pain doctors for medication, and there was not a significant difference in the incidence of adverse drug events between the groups, indicating that internet hospitals are a feasible way to guide patients with chronic pain in medication management and are not inferior to traditional face-to-face hospitals. In addition, most patients of internet hospitals were very satisfied, while patients of physical hospitals were just satisfied, which may have been due to travel fatigue, a long queue, crowded treatment areas, and time spent in the process of physical hospital treatment.

With internet technology as the carrier, one of the greatest advantages of internet hospitals is that patients can be treated at home, which further reduces the cost of patient travel, accommodation, and meals. A previous systematic review and meta-analysis found that, in terms of cost-effectiveness, 39% of studies determined that telemedicine was more cost-effective [18]. Our study revealed that the total expenses for patients of internet hospitals were lower than that for patients of physical hospitals. Although there was a reduction in nonmedical costs for patients, internet hospitals cannot provide patients with physiotherapy, nerve blocking treatment, or other similar treatments and can only adjust medication for patients. This further indicates that the current internet hospital model is suitable for patients with mild pain, stable conditions, and for follow-up visits. However, for the baseline comparison, we found that patients in the internet hospital group had higher NRS scores than those in the physical hospital group, possibly because patients could see a doctor immediately when their pain worsened via internet hospitals, while physical hospitals required appointments that may have occurred when pain levels were not at their peak.

At present, the treatment of chronic pain is mainly based on multimodal analgesia. Since an internet hospital is a carrier of multimodal analgesia for chronic pain, one of its advantages is that it can be visited anytime and anywhere through a variety of smart devices, and internet hospitals can be combined with other digital medical technologies, such as wearable devices, for at-home follow-ups. The implementation of multimodal analgesia by internet hospitals is mainly through the following: first, similar to traditional offline treatment, the patients have a web-based follow-up to assess their conditions, so as to adjust the use of medication; second, jointly with the psychological clinic for patients to conduct psychological counseling, patients are taught how to face pain and how to use mindfulness therapies; third, according to the patients’ conditions, the patients are given offline physiotherapy or invasive intervention treatment appointments at appropriate times, and those with serious conditions can directly apply for web-based admission procedures. In conclusion, the current internet hospital as a platform or a new management implementation pathway, can help patients and doctors maintain a long-term relationship, which is conducive to the stable implementation of treatment.

This study is the first to explore and verify the effectiveness of internet hospitals for managing chronic pain and our findings will help promote the use of internet hospitals for outpatient chronic pain management. Internet hospitals can be used as a part of the standardized closed-loop management of chronic pain, but they are still in the development stage for chronic disease management and cannot actively intervene in patients’ conditions or carry out continuous disease monitoring. In the future, more chronic disease management models need to be further developed for different chronic diseases.

### Limitations

This study had some limitations. First, this was a retrospective cohort study, and relevant scales and data were obtained through telephone interviews with patients, which may have led to a recall bias and cognitive biases. Second, we did not collect information on pain severity, pain location, and the types of drug use before matching, so PSM did not use this information as a covariate, which may have affected the reliability of some of the results. Meanwhile, we did not collect the time interval between patients’ visits, which may have had an impact on treatment effectiveness. In addition, the patients in the internet hospital group could only receive drug treatment through the internet, while some patients in the physical hospital group could receive both drug treatment and non–drug treatment, and we did not exclude these patients, which may have affected the comparisons for treatment effects. Furthermore, this study selected only patients who visited the pain outpatient department of the West China Hospital of Sichuan University from September 2021, to February 2023. Although the West China Hospital is the most representative hospital in southwest China, this was still a single-center study and may lack representativeness.

### Conclusions

Both physical hospitals and internet hospitals could improve the QOL for patients with chronic pain. Meanwhile, patients using internet hospitals had a greater satisfaction and lower costs. Therefore, the internet hospital can be used as one of the multichannel ways of chronic pain management outside of the hospital.

## Abbreviations

BPI: Brief Pain Inventory
D-value: quality of life at the last visit minus quality of life at the first visit
NRS: numerical rating scale
PSM: propensity score matching
QOL: quality of life
SMD: standardized mean difference
